# Macronutrient Composition and Food Form Affect Glucose and Insulin Responses in Humans

**DOI:** 10.3390/nu10020188

**Published:** 2018-02-08

**Authors:** Shila Shafaeizadeh, Leilani Muhardi, Christiani Jeyakumar Henry, Bert J. M. van de Heijning, Eline M. van der Beek

**Affiliations:** 1 Nutricia Research, Matrix Building #05-01b, 30 Biopolis Street, Singapore 138671, Singapore; 2 Danone Early Life Nutrition, Cyber 2 Tower, 15th Floor, Jl. HR. Rasuna Said #X-5 No. 13, South Jakarta 12950, Indonesia; Leilani Muhardi@danone.com; 3 Clinical Nutrition Research Centre (CNRC), Singapore Institute for Clinical Sciences (SICS), Agency for Science, Technology and Research (A*STAR), and National University Health System, Centre for Translational Medicine, 14 Medical Drive #07-02, MD 6 Building, Yong Loo Lin School of Medicine, Singapore 117599, Singapore; Jeya_Henry@sics.a-star.edu.sg; 4 Department of Biochemistry, National University of Singapore, 8 Medical Drive, Singapore 117596, Singapore; 5 Nutricia Research, Uppsalalaan 12, 3584 CT Utrecht, The Netherlands; Bert.vandeheijning@danone.com (B.J.M.v.d.H.); Eline.vanderbeek@danone.com (E.M.v.d.B.); 6 Department of Pediatrics, University Medical Centre Groningen, CA84, Room Y2.115, Hanzeplein 1, 9713 GZ Groningen, The Netherlands

**Keywords:** glycaemic index, insulinemic index, glycaemic and insulin responses, carbohydrate quality, protein quality, nutritional formula

## Abstract

Glycaemic index (GI) is used as an indicator to guide consumers in making healthier food choices. We compared the GI, insulin index (II), and the area under the curve for blood glucose and insulin as glucose (GR) and insulin responses (IR) of a newly developed liquid nutritional formula with one commercially available liquid product with different types of carbohydrates. We then evaluated the glucose and insulin responses of two test foods with comparable energy density and protein percentage but presented in different food forms (liquid vs. solid). Fourteen healthy women participated in the study. GI, II, GR, and IR were assessed after (independent) consumption of two liquid products and a solid breakfast meal. The two liquid foods showed comparable GI, whilst the liquid form appeared to produce lower median GI (25 vs. 54), and II (52 vs. 98) values compared to the solid breakfast (*p* < 0.02). The median GR and IR for solid breakfast were respectively 44% and 45% higher compared to the liquid product (*p* < 0.02). Liquid formulas with different carbohydrate qualities produced comparable glucose responses, while foods with comparable energy density and protein percentage but different food form elicited differential effects on GI, II, GR, and IR. Nutrient quality and food form need to be taken into consideration when developing low GI products to manage glycaemic responses.

## 1. Introduction

Carbohydrates are an essential part of our diet, contributing to approximately half of our total daily caloric intake [1]. However, not all carbohydrates are absorbed at the same rate. The quality of carbohydrates depends on their digestibility, absorption rates, and metabolic consequences [2]. Intrinsic carbohydrates present naturally as an integral constituent of whole fruit, vegetable and milk products, and are examples of good quality carbohydrates [3]. Excessive intake of simple carbohydrates, such as the more refined sugars in high fructose corn syrup, table sugar, and honey, has been linked to increased metabolic health risks [1,3,4,5]. Therefore, various dietary guidelines advise to reduce the consumption of simple carbohydrates and to consume better quality carbohydrates [1,6].

The glycemic index (GI) concept, originally articulated by Jenkins et al., is a simple way of characterizing carbohydrates based on their impact on postprandial glycaemic responses [7]; pure glucose represents the “standard” and is equivalent to a GI of 100. Carbohydrates with a low GI value (below 55) are more slowly digested, absorbed, and metabolised, and lead to a lower and/or slower rise in blood glucose than those with a higher GI [7]. Recent studies have shown that consumption of a low GI diet improves blood glucose control by lowering the levels of fasting blood glucose, haemoglobin A1C and urinary C-peptide, regulates body weight and promotes appetite control [8,9,10,11,12]. People with postprandial hyperglycaemia, such as pre-diabetes or impaired insulin sensitivity, are advised to consume low GI foods [8,13] and a low GI diet has been recommended to reduce the risk of type 2 diabetes mellitus (T2DM) and obesity [8,10].

The Food and Agriculture Organization (FAO) and World Health Organisation (WHO) recommend substituting high GI foods with low GI alternatives [1]. Food standard agencies in Australia, New Zealand, the UK, Indonesia, and South Africa allow the GI as a health claim [14,15,16], and the presence of a “low GI” logo on the food label [2,15,17] assists health-conscious consumers in choosing healthier packaged food.

In light of these recommendations and claims, the food industry has focused on developing low GI nutritional formulas or calorie replacers with complete nutritional profiles in different food forms [18,19]. Although liquid nutritional formulas are frequently used by consumers, information on their metabolic consequences, such as GI and II, are limited and rarely compared to solid food [12]. Both nutrient composition and food form are suggested to be important in influencing the endocrine/metabolic effects of these products [20].

Using GI as a sole indicator to define “healthier choice” products has several limitations. Glucose response (GR) is influenced by multiple factors including carbohydrate quality, nutrient composition, energy density, insulinemic response, acid levels, processing and cooking methods, and different food forms [10,21]. The insulin index (II) captures the amount of insulin released in response to a certain, fixed carbohydrate load in a particular food [21,22]. Hence, measuring the II, in addition to the GI, would provide more comprehensive metabolic information.

To our knowledge, only a few studies [23,24,25] have attempted to determine both the GI and II values of nutrient-enriched liquid products with carbohydrates that differ in type and quality. Thus, our first aim was to compare the GI, II, GR, and insulin response (IR) of a newly developed product with improved carbohydrate quality against a commercially available liquid product. Since limited information is available on the influence of food form on GI and II values, the second aim of the study was to evaluate the GI and II of two test food products with comparable energy density and protein composition but presented as two different forms, namely solid and liquid.

## 2. Materials and Methods

### 2.1. Participants

The inclusion of 10 participants provides a reasonable degree of power and precision for measuring GI and is the usual acceptable size to investigate GI. However, the number of participants can be increased if the aim of the study is to detect small differences in GI or when greater precision is required [26]. Therefore, this study enrolled 14 healthy Singaporean women (nine Chinese, four Malay, one Indian) within the age range of 21–40 years who fulfilled the inclusion criteria: no diabetes mellitus, no food allergies or intolerance, no major medical/surgical events, and no medication use e.g., steroids, protease inhibitors, or antipsychotics. The study was conducted at the Temasek Applied Science Research Centre, a GI-accredited research unit, and in accordance with the Declaration of Helsinki. The protocol was approved by the Parkway Independent Ethics Committee (PIEC/2013/029), and informed consent was obtained from all study participants prior to starting the study.

### 2.2. Study Design

The study was a randomized, controlled crossover design, whereby each participant returned for six different test days over a six-month period. Before each test session, all study participants were advised to eat a regular evening meal followed by a 10–14 h overnight fast. Participants were requested not to consume alcohol before or during the study day and to refrain from intense physical activity.

On the test days, participants consumed either the reference food, a 50 g glucose drink as the reference food (dextrose anhydrous dissolved in 250 mL plain water) or one of the liquid test foods or solid meal breakfast in random order. There was a minimum one-day wash out period between study days.

### 2.3. Test Foods

Three references and three test meals were tested, each containing 50 g of carbohydrates. The test foods were a liquid concept product (CP), a commercially available liquid product (CAP), and a high protein breakfast meal (BFM). CP and CAP differed in carbohydrate composition and macronutrient contribution to total energy. The macronutrient breakdown and the type of carbohydrate in each test food are summarized in Table 1.

The CP is a newly formulated liquid drink enriched in specific micronutrients and long chain poly unsaturated fatty acids (LC-PUFAs), designed as a total or partial calorie meal replacer intended for pregnant women. The carbohydrates in this product were a combination of lactose, slowly digestible carbohydrates and isomaltulose, with a combination of whey and soy as the main sources of protein. The CAP is a commercially available liquid milk-based product intended for pregnant women. The main sources of carbohydrate in the CAP were lactose and sucrose, and the main sources of protein were whey and casein. The CAP is available as powder, is intended for use in between or accompanying a meal, and is made into a liquid by adding water according to instructions on pack. The BFM consisted of 85 g fine-grain whole meal bread, 260 mL Emborg skimmed milk, and one hard-boiled egg, providing approximately 24 g protein.

### 2.4. Measurement of GR and IR and Determination of GI and II

Following a brief rest at the start of each session, two baseline finger prick blood samples were taken, at a five min interval, to measure fasting glucose and insulin levels. Test foods or glucose drink were given directly after the second finger prick (time 0) and were to be consumed within 14 min, together with 250 mL plain water. Subsequent finger pricks were obtained at 15, 30, 45, 60, 90, and 120 min after time 0. At each time point, capillary blood was collected for glucose analysis (100 μL) and insulin analysis (300 μL). Each food was tested only once, whereas the glucose drink was tested three times in each subject, on separate days, across the whole study, in line with GI testing protocol recommendations [27].

Blood samples were centrifuged, and plasma was transferred into an uncoated tube for same-day analysis of glucose (in duplicate) using a glucose-hexokinase enzymatic assay on a spectrophotometric analyser with internal controls (Calibrate YSI 2300 Stat PLUS Glucose & Lactate analyser, YSI Life Sciences, Tunbridge Wells, UK). A 2 h GR curve was generated and the incremental area under the curve for glucose (AUCG) responses was determined according to the trapezoidal method. Any area below the baseline fasting value was ignored. The calculated median of AUCG for three test foods from 14 participants was compared with the response to reference glucose drink (median of three measurements), and the GI value of glucose solution set as 100. The median GI of each food was calculated using the following equations [27].

$$\mathrm{GI}\;\mathrm{value}\;\mathrm{for}\;\mathrm{each}\;\mathrm{test}\;\mathrm{food}\;=\frac{\mathrm{AUCG}\;\mathrm{value}\;\mathrm{for}\;\mathrm{the}\;\mathrm{test}\;\mathrm{food}}{\mathrm{AUCG}\;\mathrm{value}\;\mathrm{for}\;\mathrm{the}\;\mathrm{reference}\;\mathrm{food}\;}\times 100$$

The level of insulin at each time point was analysed (in duplicate) using an Amplified Luminescent Proximity Homogenous Assay technology (AlphaScreen^®^, PerkinElmer Inc., Waltham, MA, USA) human insulin detection assay (AlphaLISA^®^ Human Insulin Immunoassay Kit, PerkinElmer Inc., Waltham, MA, USA) according to the manufacturer’s instructions. Plasma insulin was plotted across 2 h to generate a curve, the median incremental area under the curve for insulin (AUCI) was calculated for 14 participants, then compared with the response to the reference glucose drink (set at 100). The median II value for each test food was calculated using following equation [26,27].

$$\mathrm{II}\;\mathrm{value}\;\mathrm{for}\;\mathrm{each}\;\mathrm{test}\;\mathrm{food}\;=\frac{\mathrm{AUCI}\;\mathrm{value}\;\mathrm{for}\;\mathrm{the}\;\mathrm{test}\;\mathrm{food}}{\mathrm{AUCI}\;\mathrm{value}\;\mathrm{for}\;\mathrm{the}\;\mathrm{reference}\;\mathrm{food}\;}\times 100$$

### 2.5. Nutrient Composition

The two liquid test foods and one solid meal delivered energy ranging from 322 to 444 kcal based on 50 g available carbohydrate (Table 1).

The CP contained 208 kcal per 200 mL (recommended daily serving size) with a mixture of slow-release carbohydrates (lactose, isomaltulose, and slowly digestible starch) and a mixture of both animal and plant proteins. The CP had a lower percentage of carbohydrate (45 Energy%) and protein (18.8%) but higher percentage of fat (33 Energy%, of which 29% was PUFA) compared to CAP.

As the composition of the CAP was more comparable to the BFM, with regards to contribution of protein to total energy (~26.5 Enegry%), energy density (3.7 vs. 3.5) and carbohydrate (56% vs. 62%), we chose to evaluate the independent role of food form, liquid vs. solid, on GI, II, GR, and IR by comparing CAP to BFM. The GI value was calculated based on the nutritional facts on the labels of the CAP and components of the BFM. Therefore, it does not give information regarding the exact amount of different types of carbohydrate (lactose, starch, and sucrose), lipids, and proteins in the test products.

### 2.6. Statistical Analysis

The Shapiro-Wilk test was applied to compare the normally distributed set of values for the GI, II, AUCG and AUCI. The null hypothesis that “sample distribution is normal” was rejected as the *p* value was >0.05. Therefore, the GI, II, AUCG, and AUCI were presented as median ± interquartile range (IQR). If a value was outside the range of median ± 3.0 IQR, it was classified as an extreme outlier.

The non-parametric Wilcoxon signed rank test was applied due to the wide distribution of data in each group and because the mean ± standard deviation (SD) were not robust against extreme outliers. The Wilcoxon signed rank test was used to evaluate the differences between the medians for CAP vs. CP or CAP vs. BFM, for total GI, II, AUCI, and AUCG, as well as at all time points (0, 15, 30, 45, 60, 90, 120 min).

As several Wilcoxon tests were applied on the same dataset simultaneously, the tests were subject to the rules of probability. Some of the tests could display significant results by pure chance, therefore, the level of significance was corrected according to the method described by Benjamini Hochberg (Table S1). The *p*-values were calculated by Wicoxon tests on the same data, together with the desired level of significance (*p* = 0.05). The corrected significance level was equal to *p* = 0.0228 [28]. SPSS Version 22.0 (IBM SPSS Statistics 22.0, New York, NY, USA) was used for all statistical analyses and the corrected *p* values were considered statistically significant at *p* < 0.0228. Box plots graphs in SPSS were used to show the difference between glucose and insulin responses between CP and CAP, and CAP and BFM, at all time points (0, 15, 30, 45, 60, 90, 120 min).

## 3. Results

Study participants were between 21 and 27 years old, with a mean age of 24 ± 1.93 years (mean ± SD). Their body mass index (BMI) ranged from 19.7 to 25.2 kg/m^2^, with a mean BMI of 22.4 ± 2.34 kg/m^2^ (mean ± SD).

### 3.1. Comparison of GI, II, GR, and IR for Two Liquid Foods

**Figure 1:**
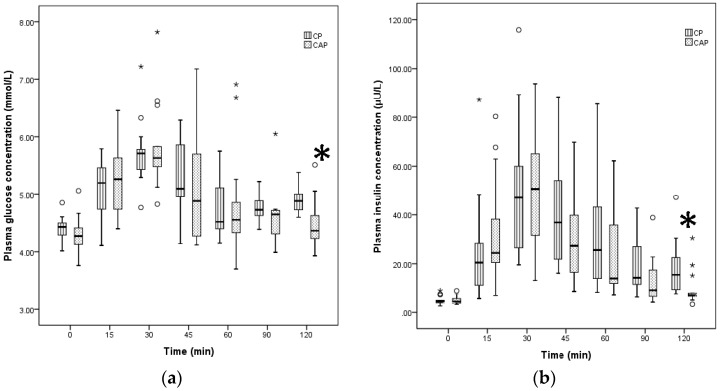
Plasma glucose (**a**) and insulin (**b**) after ingestion of two liquid test foods. CP = concept product; CAP = commercially available product; Benjamini Hochberg with corrected levels of significance *p* = 0.0228; * extreme outliers (median ± 3.0 interquartile range (IQR)); ° inner-fence or minor outliers (median ± 1.5 IQR). Differences in the median of glucose and insulin responses between CAP and CP at 120 min * *p* < 0.02, based on non-parametric Wilcoxon signed rank test.

Both liquid foods, CP and CAP, were classified as low GI (GI < 55). Based on the Wilcoxon signed rank test, the median GI of CP was not significantly different from the median GI of CAP (Table 2). Both CP and CAP reached peak blood glucose at approximately 30 min; this was followed by a gradual decline, resulting in identical time courses of blood glucose for the two liquid nutritional formulas (Figure 1). There were no significant differences in GR between CP and CAP (Table 2.)

The median II values and IR for CP were not significantly different from those for CAP. The timing of insulin peak for both liquid test foods was comparable at 30 min after intake. There was no significant difference between insulin values of CP at different time points compared to CAP insulin response. At 120 min, the median glucose and insulin responses were higher for CP compared to CAP, but there was a higher number of outliers related to CAP consumption (Figure 1). Overall, the GI, II, GR, and IR assessment revealed mostly similarities between responses to CAP and CP (Table 2).

### 3.2. Comparison of the GI, II, GR, and IR for Liquid vs. Solid Foods

The liquid CAP and solid BFM, comparable only in energy density and protein percentage, were both classified as low GI. However, the GI and GR for CAP, the liquid, were significantly lower than for BFM, the solid meal (*p* < 0.02, Table 2). 

**Figure 2:**
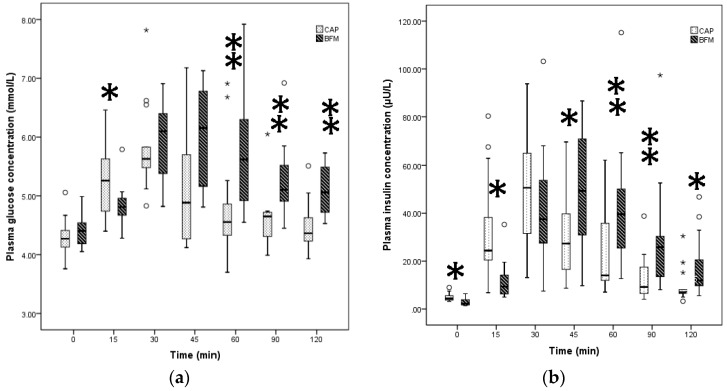
Plasma glucose (**a**) and insulin (**b**) after ingestion of test foods with different food forms. BFM = breakfast meal; CAP = commercially available product; Benjamini Hochberg with corrected levels of significance *p* = 0.0228 was applied; * extreme outliers (median ± 3.0 interquartile range (IQR); ° inner-fence or minor outliers (median ± 1.5 IQR); (**a**) Differences in the median of glucose responses between CAP and BFM at 15 min * *p* < 0.02, and at 60, 90 and 120 min ** *p* < 0.005, based on non-parametric Wilcoxon signed rank test; (**b**) Differences in the median of insulin responses between CAP and BFM at 0, 15, 45, 120 min * *p* < 0.02, and at 60 and 90 min ** *p* < 0.005, based on non-parametric Wilcoxon signed rank test.

CAP, the liquid food, produced an early rise in plasma glucose concentration with the highest median level around 30 min whereas the solid food showed a later peak for blood glucose, i.e., 45 min after consumption (Figure 2). The solid food produced significantly lower blood glucose values at 15 min after ingestion compared to CAP (*p* < 0.02), whereas the levels of blood glucose induced by CAP were significantly lower at 60, 90, and 120 min after ingestion when compared to those induced by BFM (*p* < 0.005, Figure 2).

CAP, the liquid food, also resulted in a significantly lower II and IR compared to solid food (*p* < 0.02). IR for CAP peaked at 30 min but peaked at 45 min for solid food. The solid food induced significantly lower insulin values at 15 min compared to the liquid but significantly higher levels of insulin at 45, 60, 90, and 120 min compared to CAP (*p* < 0.02, Figure 2).

## 4. Discussion

All of our included test foods were classified as low GI. Two liquid nutritional formulas with different types of carbohydrate produced comparable glucose and insulin responses, although consumption of the CP formula appeared to result in fewer outliers. This suggests that it is not possible to estimate GI from the nutritional composition alone and reaffirms the need to conduct human studies whilst taking into account that responses may differ considerably between participants. The liquid products differed considerably in macro- and micronutrient composition. When the two test foods with comparable percentage of protein and energy density but differing food form (CAP and BFM) were compared, the liquid form elicited significantly lower GI and II values, as well as lower GR and IR.

The present study showed that the addition of more slowly digestible starch, fibre, and fat in the CP did not improve the GI of the product compared to CAP. The tested liquid products differed with regard to protein composition and the contribution of protein (and fat) to the total energy provided. This further highlights the fact that other macronutrients may be relevant to GI.

Although the median GR and IR were not significantly different between two liquid test foods, the interquartile range was narrower for CP compared to CAP, suggesting that the consumption of CP resulted in less individual variability compared to CAP. Thus, both carbohydrate and protein quality may be relevant to improving consistency in GR and IR results between participants.

The significant impact of macronutrient composition, food form and viscosity on GI and II values have been reported previously [21,29,30,31]. Insulin release can be affected by specific amino acids, as they can directly and indirectly (via incretin release) stimulate insulin secretion [32,33,34]. In particular, the amino acid profile of dairy proteins may be uniquely able to synergistically stimulate insulin secretion and to promote a high anabolic insulin response [35] resulting in insulin secretions quite disproportional to carbohydrate content [32,36]. This may be one of the explanations why the BFM, a meal consisting of bread, milk, and egg, had a higher II value compared to the CAP [37].

Carbohydrate quality is one of the significant determinants of glucose and insulin metabolism. Reaven et al. reported that plasma glucose and insulin responses to 50 g starch were significantly lower than in response to sucrose in both liquid and solid form [38]. In our study, the carbohydrate fraction of CAP consisted of sucrose (1:1 M of glucose and fructose) and lactose (1:1 M of glucose and galactose) from cow’s milk, whilst the carbohydrates in the BFM were starch from wholemeal bread and lactose from cow’s milk. Alone, the GI values of these carbohydrates are 37 ± 4 for milk, 43 for lactose, 74 ± 2 for wholemeal bread, and 65 ± 4 for sucrose (variations are based on different brands) [39]. However, in our study, the median GI values of the liquid and solid foods were lower, approximately 25.5 and 54.5, respectively; these numbers are tending toward the GI of milk, and it seems it has not affected a lot by the presence of sucrose and starch components. The presence of milk in the BFM may have attenuated the GI and GR. This is in line with a study done by Li et al., which reported lower GR to 240 mL milk compared to 240 mL sugar-sweetened coffee in the context of a solid standard breakfast (ham and scrambled egg sandwich and jelly) [40]. However, in our study, the GI and GR as AUCG were still significantly higher in response to the solid food compared to the liquid. Thus, the differences in GI and GR values of CAP and BFM may not be entirely driven by carbohydrate quality; other components in the foods may influence the GI values. As the quality of protein is similar in both test foods, it seems that food forms can play an influential role.

Previous studies have shown food form can influence nutrient bioavailability and metabolic consequences, such as plasma insulin, glucose, cholecystokinin, ghrelin and glucagon-like peptide-1, differently [41]. Stenvers and colleagues compared the effect of a three-month intake of a liquid formula (high in monounsaturated fatty acids, fructose and fiber) with an isoenergetic solid breakfast (288 vs. 292 kcal) on GR and IR in 20 participants with T2DM; they observed a reduction in GR and IR after the three-month trial [42].

Wang and colleagues also reported that higher intake of added sugars (10 g/day) from only liquid foods was linked to higher fasting glucose and fasting insulin, as well as increased insulin resistance. No associations were shown with intake of added sugars from solid sources [43]. A study among older adults (>60 years) indicated that nutrient-matched nutritional supplements had different metabolic responses when given in either solid or liquid form. Plasma glucose and insulin responses were lower after ingestion of a liquid beverage compared to a solid food [44]. Finally, a study by Edes et al. compared the metabolic effects of a liquid nutritional formula and a solid standard test meal on GI, GR, and IR among 12 participants and reported that GI and GR did not differ between isocaloric liquid and solid test meal [13].

Differences in energy density and macronutrient composition between liquids and solids can also influence GR and IR [45]. In the present study, the CAP had a comparable energy density to BFM but elicited lower GI and II values. The CAP also resulted in earlier peak glucose and insulin compared to BFM. Indeed, GR and IR seem to be influenced by gastric emptying time and rate of absorption, and food viscosity could be a factor that influences glucose-insulin responses. A low viscosity oat bran beverage resulted in significantly higher plasma glucose and insulin when compared with a high-viscosity oat bran beverage, implying that viscosity can modulate glucose-insulin metabolism [46]. The rate of gastric emptying varies depending on food form and/or viscosity [41,47,48] and may account for up to 35% of the variance in glycaemic control [43,49,50].

The present study has made two important observations. First, a reduction in GR from a low GI food may be achieved by altering carbohydrate and/or protein quality by using slowly digestible starch e.g., isomaltulose or insulinotropic disaccharides such as lactose, and insulinotropic proteins such as soy, whey, and casein. Since these ingredients have independent and additive effects on glycaemic response, it is impossible to predict their individual impact on glycaemia without performing human studies. Second, foods with comparable protein and energy content in either liquid or solid form appear to differ in GI and II response, with the liquid form resulting in a lower GI and II response. Taken together, these observations provide two practical outcomes.

If food form can impact glucose-insulin metabolism, it is essential to investigate the short- and long-term effects of low GI liquid test foods with different viscosities on glycaemic and insulin profiles, particularly in people with compromised glycemic control. In the current study, liquid and solid foods were matched on the basis of protein and energy density. Future studies should focus on the independent effect of food form on GI and II by considering identical food composition and food viscosity.

Given that food form (i.e., liquid or solid) had such an impact on GI and II, it would be instructive to determine if milk was removed from the context of solid breakfast or if the liquid foods was transformed into a gel, whether the solid meal (BFM) would still maintain its lower GI and II status compared to the liquid test food.

This will enable us to develop foods with optimal nutrient composition and agreeable organoleptic properties for people that could benefit from a low GI diet such as the elderly and people at risk for the developing T2DM. The latter is relevant for people with obesity, as well as those with impaired glucose tolerance or pregnant women with gestational diabetes mellitus (GDM). In addition, these foods should be tailored to the specific nutrient needs and overall diets of these target populations.

## 5. Conclusions

Nutrient composition and food form appear to influence both GR and IR. Although several nutrients, notably proteins, slowly digesting carbohydrates and fibre may all be used to reduce glycaemia, it is impossible to predict their effects on glycaemic response without conducting human trials. A combination of GI values with other indices such as II may be better biomarkers to facilitate selection of “healthier choice” food products. For foods with comparable nutrient composition, the liquid form appears to produce a lower GI and II. Given that both nutrient composition and food form (i.e., liquid or solid) have an impact on GI and II, future research should focus on developing foods with optimal nutrient composition and agreeable organoleptic properties and forms for those people who need to manage hyperglycaemia and are prone to T2DM, such as women exposed to GDM during pregnancy [51].

## Figures and Tables

**Table 1:** Nutrient composition of test foods based on 50 g available carbohydrate.

Test Food ^1^	Food Form ^2^	Total Calorie (kcal/100 g or mL)	CHO (g and %) ^3^	CHO Type	Protein (g) ^3^	Protein Type	Fat (g) ^3^	Fiber (g)	Daily Serving Size (mL)
RF	50 g + 250 mL water	200	50 (100%)	Dextrose anhydrous	-	-	-	-	-
CP	427 mL beverage	444 (104)	50 (45%)	Lactose Isomaltulose Slowly digestible starch	20.9 (18.8%)	Soy Whey casein	16.2 (32.8%)	8.5	200
CAP	86.2 g powder + 346 mL water	322 (374)	50 (62.1%)	Lactose Sucrose	21.5 (26.7%)	Whey casein	3.9 (11%)	0	230
BFM	85 g wholemeal bread + 260 mL skim milk + 35 g boiled egg	356 (356)	50 (56.2%)	Starch Lactose	23.4 (26.3%)	Gluten Whey casein	6.4 (16.20%)	5.1	

^1^ RF = reference food (glucose drink); CP = concept product; CAP = commercially available product; BFM = breakfast meal, CHO = carbohydrate. ^2^ All test meals were served immediately with 250 mL of plain water. ^3^ the amount of protein, fat and fiber (g) are as the percentage of total energy.

**Table 2:** Peak and area under the curve (AUC) values for blood insulin and glucose and GI/II values for reference and test foods.

Test Foods	Peak Glucose (mmol/L)	AUCG (mmol /L)	GI	Peak Insulin (µU/L)	AUCI (µU/L)	II
Reference food	7.62 (1.23)	203.38 (68.11)	100	52.13 (34.48)	2881.03 (3401.76)	100
CP	5.70 (0.43)	68.65 (38.18)	29.50 (8.00)	47.05 (40.79)	2189.7 (1514.75)	71.50 (27.00)
CAP	5.63 (0.58)	65.50 (61.05) ^a^	25.50 (19.75) ^a^	50.66 (35.93)	1781.6 (1817.00) ^a^	52.50 (40.25) ^a^
BFM	6.15 (1.68)	117.50 (55.53) ^b^	54.50 (27.50) ^b^	49.37 (41.48)	3254.35 (2534.83) ^b^	98 (70.00) ^b^

Reference food = glucose drink; CP = concept product; CAP = commercially available product; BFM = breakfast meal; AUCG = Area under the curve for blood glucose; GI = Glycaemic index; AUCI = Area under the curve for blood insulin; II = Insulin index. Values were calculated from 14 participants and are expressed as median (interquartile range). Extreme outliers = median ± (3.0 IQR). Difference between CAP and BFM (a vs. b, *p* < 0.02) based on non-parametric Wilcoxon signed rank test.

## Supplementary Materials

The following are available online at http://www.mdpi.com/2072-6643/10/2/188/s1. Table S1: The correct level of significance according to the method described by Benjamini Hochberg.

## References

[1] Food and Agriculture Organization (FAO), World Health Organisation (WHO) (1998). Carbohydrates in Human Nutrition: Report of a Joint FAO/WHO Expert Consultation. FAO Food Nutr. Pap..

[2] Sentko A. (2013). Innovative low-glycaemic carbohydrates: An update. Nutrafoods.

[3] Howard B.V., Wylie-Rosett J. (2002). Sugar and cardiovascular disease: A statement for healthcare professionals from the committee on nutrition of the council on nutrition, physical activity, and metabolism of the American Heart Association. Circulation.

[4] Ruxton C.H. (2003). Dietary guidelines for sugar: The need for evidence. Br. J. Nutr..

[5] Johnson R.K., Appel L.J., Brands M., Howard B.V., Lefevre M., Lustig R.H., Sacks F., Steffen L.M., Wylie-Rosett J. (2009). Dietary sugars intake and cardiovascular health: A scientific statement from the American Heart Association. Circulation.

[6] Bornet F.R., Alamowitch C., Slama G. (1995). Methods to assess glucose metabolism in humans in relation to carbohydrate and fibre in the diet. Eur. J. Clin. Nutr..

[7] Ajala O., English P., Pinkney J. (2013). Systematic review and meta-analysis of different dietary approaches to the management of type 2 diabetes. Am. J. Clin. Nutr..

[8] Erbe J.K. (2009). Low glycemic index diets for the management of diabetes. Am. Fam. Phys..

[9] Jenkins D.J., Wolever T.M., Taylor R.H., Barker H., Fielden H., Baldwin J.M., Bowling A.C., Newman H.C., Jenkins A.L., Goff D.V. (1981). Glycemic index of foods: A physiological basis for carbohydrate exchange. Am. J. Clin. Nutr..

[10] Ludwig D.S., Majzoub J.A., Al-Zahrani A., Dallal G.E., Blanco I., Roberts S.B. (1999). High glycemic index foods, overeating, and obesity. Pediatrics.

[11] Alfenas R.C., Mattes R.D. (2005). Influence of glycemic index/load on glycemic response, appetite, and food intake in healthy humans. Diabetes Care.

[12] Ball S.D., Keller K.R., Moyer-Mileur L.J., Ding Y.W., Donaldson D., Jackson W.D. (2003). Prolongation of satiety after low versus moderately high glycemic index meals in obese adolescents. Pediatrics.

[13] Edes T.E., Shah J.H. (1998). Glycemic index and insulin response to a liquid nutritional formula compared with a standard meal. J. Am. Coll. Nutr..

[14] Wolever T.M. (2013). Glycemic index claims on food labels: Review of Health Canada’s evaluation. Eur. J. Clin. Nutr..

[15] Aziz A., Dumais L., Barber J. (2013). Health Canada’s evaluation of the use of glycemic index claims on food labels. Am. J. Clin. Nutr..

[16] Hoefkens C., Verbeke W. (2013). Consumers’ health-related motive orientations and reactions to claims about dietary calcium. Nutrients.

[17] Buul J.V., Brouns F.J. (2015). Nutrition and health claims as marketing tools. Crit. Rev. Food Sci. Nutr..

[18] Li L., Xu J., Zhu W., Fan F., Bai Q., Huang C., Liu J., Li Z., Sederholm M., Norstedt G. (2016). Effect of a macronutrient preload on blood glucose level and pregnancy outcome in gestational diabetes. J. Clin. Transl. Endocrinol..

[19] Tey S.L., Van Helvoort A., Henry C.J. (2016). Glycaemic responses to liquid food supplements among three Asian ethnic groups. Eur. J. Nutr..

[20] Comerford K.B., Pasin G. (2016). Emerging evidence for the importance of dietary protein source on glucoregulatory markers and type 2 diabetes: Different effects of dairy, meat, fish, egg, and plant protein foods. Nutrients.

[21] Holt S.H., Miller J.C., Petocz P. (1997). An insulin index of foods: The insulin demand generated by 1000-kJ portions of common foods. Am. J. Clin. Nutr..

[22] Venn B.J., Green T.J. (2007). Glycemic index and glycemic load: Measurement issues and their effect on diet-disease relationships. Eur. J. Clin. Nutr..

[23] Brand-Miller J., Atkinson F., Rowan A. (2013). Effect of added carbohydrates on glycemic and insulin responses to children’s milk products. Nutrients.

[24] Li D., Zhang P., Guo H., Ling W. (2014). Taking a low glycemic index multi-nutrient supplement as breakfast improves glycemic control in patients with type 2 diabetes mellitus: A randomized controlled trial. Nutrients.

[25] Wright C.J., Atkinson F.S., Ramalingam N., Buyken A.E., Brand-Miller J.C. (2015). Effects of human milk and formula on postprandial glycaemia and insulinaemia. Eur. J. Clin. Nutr..

[26] Brouns F., Bjorck I., Frayn K.N., Gibbs A.L., Lang V., Slama G., Wolever T.M. (2005). Glycaemic index methodology. Nutr. Res. Rev..

[27] Wolever T.M., Jenkins D.J., Jenkins A.L., Josse R.G. (1991). The glycemic index: Methodology and clinical implications. Am. J. Clin. Nutr..

[28] Benjamini Y., Hochberg Y. (1995). Controlling the false discovery rate: A practical and powerful approach to multiple testing. J. R. Stat. Soc..

[29] Björck I., Liljeberg H., Ostman E. (2000). Low glycaemic-index foods. Br. J. Nutr..

[30] Liljeberg H., Bjorck I. (1998). Delayed gastric emptying rate may explain improved glycaemia in healthy subjects to a starchy meal with added vinegar. Eur. J. Clin. Nutr..

[31] Liljeberg H.G., Akerberg A.K., Bjorck I.M. (1999). Effect of the glycemic index and content of indigestible carbohydrates of cereal-based breakfast meals on glucose tolerance at lunch in healthy subjects. Am. J. Clin. Nutr..

[32] Nuttall F.Q., Gannon M.C. (2004). Metabolic response of people with type 2 diabetes to a high protein diet. Nutr. Metab..

[33] Park Y.M., Heden T.D., Liu Y., Nyhoff L.M., Thyfault J.P., Leidy H.J., Kanaley J.A. (2015). A high-protein breakfast induces greater insulin and glucose-dependent insulinotropic peptide responses to a subsequent lunch meal in individuals with type 2 diabetes. J. Nutr..

[34] Brand-Miller J.C., Liu V., Petocz P., Baxter R.C. (2005). The glycemic index of foods influences postprandial insulin-like growth factor-binding protein responses in lean young subjects. Am. J. Clin. Nutr..

[35] Gunnerud U.J., Ostman E.M., Bjorck I.M. (2013). Effects of whey proteins on glycaemia and insulinaemia to an oral glucose load in healthy adults; a dose-response study. Eur. J. Clin. Nutr..

[36] Nuttall F.Q., Mooradian A.D., Gannon M.C., Billington C., Krezowski P. (1984). Effect of protein ingestion on the glucose and insulin response to a standardized oral glucose load. Diabetes Care.

[37] Nilsson M., Stenberg M., Frid A.H., Holst J.J., Bjorck I.M. (2004). Glycemia and insulinemia in healthy subjects after lactose-equivalent meals of milk and other food proteins: The role of plasma amino acids and incretins. Am. J. Clin. Nutr..

[38] Reaven G.M. (1979). Effects of differences in amount and kind of dietary carbohydrate on plasma glucose and insulin responses in man. Am. J. Clin. Nutr..

[39] Atkinson F.S., Foster-Powell K., Brand-Miller J.C., Brand-Miller J.C. (2008). International tables of glycemic index and glycemic load values: 2008. Diabetes Care.

[40] Li J., Janle E., Campbell W.W. (2017). Postprandial glycemic and insulinemic responses to common breakfast beverages consumed with a standard meal in adults who are overweight and obese. Nutrients.

[41] Zhu Y., Hsu W.H., Hollis J.H. (2013). The impact of food viscosity on eating rate, subjective appetite, glycemic response and gastric emptying rate. PLoS ONE.

[42] Stenvers D.J., Schouten L.J., Jurgens J., Endert E., Kalsbeek A., Fliers E., Bisschop P.H. (2014). Breakfast replacement with a low-glycaemic response liquid formula in patients with type 2 diabetes: A randomised clinical trial. Br. J. Nutr..

[43] Wang J., Light K., Henderson M., O’Loughlin J., Mathieu M.E., Paradis G., Gray-Donald K. (2014). Consumption of added sugars from liquid but not solid sources predicts impaired glucose homeostasis and insulin resistance among youth at risk of obesity. J. Nutr..

[44] Apolzan J.W., Leidy H.J., Mattes R.D., Campbell W.W. (2011). Effects of food form on food intake and postprandial appetite sensations, glucose and endocrine responses, and energy expenditure in resistance trained v. sedentary older adults. Br. J. Nutr..

[45] Bell E.A., Castellanos V.H., Pelkman C.L., Thorwart M.L., Rolls B.J. (1998). Energy density of foods affects energy intake in normal-weight women. Am. J. Clin. Nutr..

[46] Juvonen K.R., Purhonen A.K., Salmenkallio-Marttila M., Lahteenmaki L., Laaksonen D.E., Herzig K.H., Uusitupa M.I., Poutanen K.S., Karhunen L.J. (2009). Viscosity of oat bran-enriched beverages influences gastrointestinal hormonal responses in healthy humans. J. Nutr..

[47] Marciani L., Gowland P.A., Spiller R.C., Manoj P., Moore R.J., Young P., Fillery-Travis A.J. (2001). Effect of meal viscosity and nutrients on satiety, intragastric dilution, and emptying assessed by MRI. Am. J. Physiol. Gastrointest. Liver Physiol..

[48] Louie J.C., Markovic T.P., Ross G.P., Foote D., Brand-Miller J.C. (2013). Timing of peak blood glucose after breakfast meals of different glycemic index in women with gestational diabetes. Nutrients.

[49] Horowitz M., Edelbroek M.A., Wishart J.M., Straathof J.W. (1993). Relationship between oral glucose tolerance and gastric emptying in normal healthy subjects. Diabetologia.

[50] Marathe C.S., Rayner C.K., Jones K.L., Horowitz M. (2013). Relationships between gastric emptying, postprandial glycemia, and incretin hormones. Diabetes Care.

[51] Hod M., Kapur A., Sacks D.A., Hadar E., Agarwal M., Di Renzo G.C., Cabero Roura L., McIntyre H.D., Morris J.L., Divakar H. (2015). The international federation of gynecology and obstetrics (FIGO) initiative on gestational diabetes mellitus: A pragmatic guide for diagnosis, management, and care. Int. J. Gynaecol. Obstet..

