# Supplementary material for: Macronutrient Composition and Food Form Affect Glucose and Insulin Responses in Humans
Source: Nutrients. 2018 Feb 8;10(2):188. doi: 10.3390/nu10020188 (PMC5852764; doi:10.3390/nu10020188)
Supplement: Supplementary file 1 [file nutrients-10-00188-s001.pdf]

Table S1. The correct level of significance according to the method described by Benjamini Hochberg.

Variables[1]	*p*-values(p<0.05)	Correct *p-value* (p<0.023)
AUCG CP vs CAP	0.778	0.0431
GI CP vs CAP	0.9	0.0472
AUCI CP vs CAP	0.074	0.0319
II CP vs CAP	0.035	0.0264
0 glucose response CP vs CAP	0.067	0.0306
15 min glucose response CP vs CAP	0.221	0.0389
30 min glucose response CP vs CAP	0.826	0.0444
45 min glucose response CP vs CAP	0.177	0.0347
60 min glucose response CP vs CAP	0.861	0.0458
90 min glucose response CP vs CAP	0.055	0.0278
120 min glucose response CP vs CAP	0.002	0.01
0 insulin response CP vs CAP	0.975	0.05
15 min insulin response CP vs CAP	0.221	0.0403
30 min insulin response CP vs CAP	0.925	0.0486
45 min insulin response CP vs CAP	0.035	0.025
60 min insulin response CP vs CAP	0.158	0.0333
90 min insulin response CP vs CAP	0.064	0.292
120 min insulin response CP vs CAP	0.005	0.0167
AUCG BFM vs CAP	0.005	0.0194
GI BFM vs CAP	0.005	0.0181
AUCI BFM vs CAP	0.009	0.0236
II BFM vs CAP	0.005	0.0153
0 glucose response BFM vs CAP	0.198	0.0361
15 min glucose response BFM vs CAP	0.006	0.0236

30 min glucose response BFM vs CAP	0.51	0.0417
45 min glucose response BFM vs CAP	0.004	0.0139
60 min glucose response BFM vs CAP	0.008	0.0222
90 min glucose response BFM vs CAP	0.001	0.0014
120 min glucose response BFM vs CAP	0.001	0.0028
0 insulin response BFM vs CAP	0.001	0.0083
15min insulin response BFM vs CAP	0.002	0.0097
30 min insulin response BFM vs CAP	0.221	0.0389
45 min insulin response BFM vs CAP	0.002	0.0125
60 min insulin response BFM vs CAP	0.001	0.0028
90 min insulin response BFM vs CAP	0.001	0.0042
120 min insulin response BFM vs CAP	0.002	0.0097

[1.] CP = concept product; CAP = commercially available product; BFM = breakfast meal; AUCG = Area under the curve for blood glucose; AUCI = area under the curve for blood insulin; GI= Glycaemic index; II= Insulin index.
